# Supplementary figures and images for: Characterization of a thalamic nucleus mediating habenula responses to changes in ambient illumination
Source: BMC Biol. 2017 Oct 31;15:104. doi: 10.1186/s12915-017-0431-1 (PMC5670518; doi:10.1186/s12915-017-0431-1)

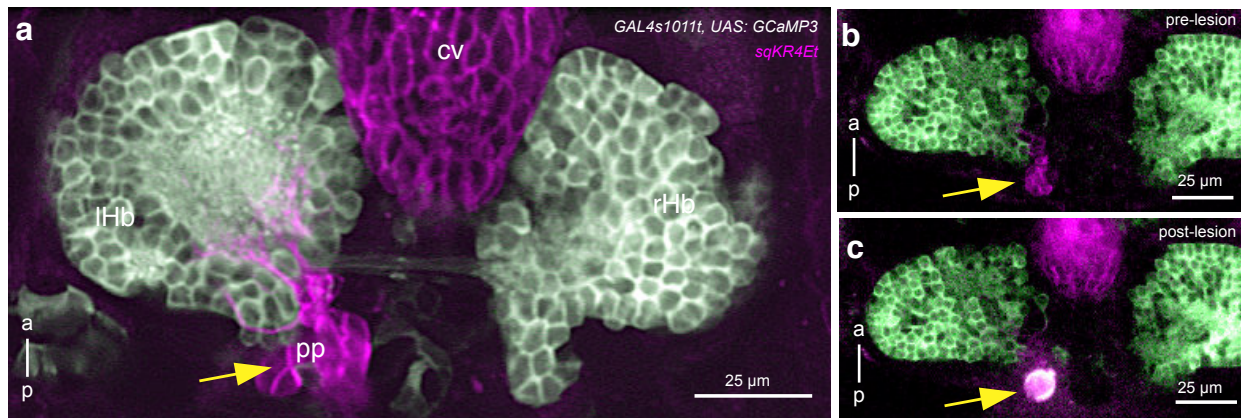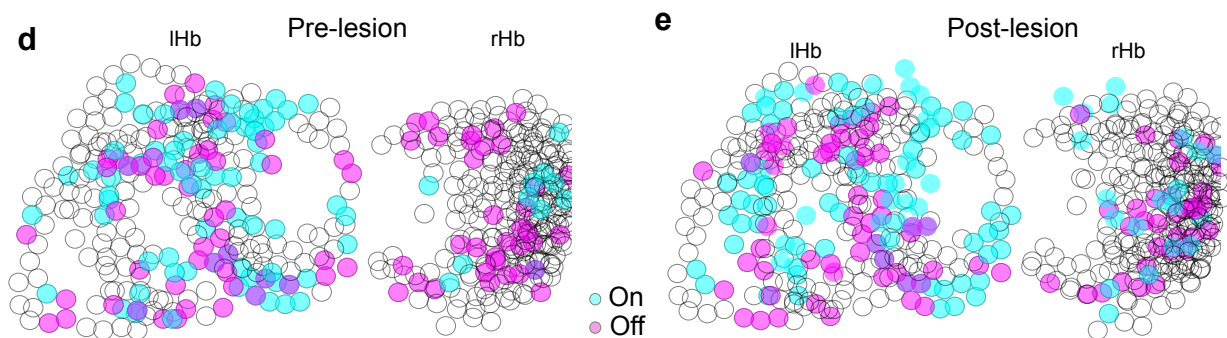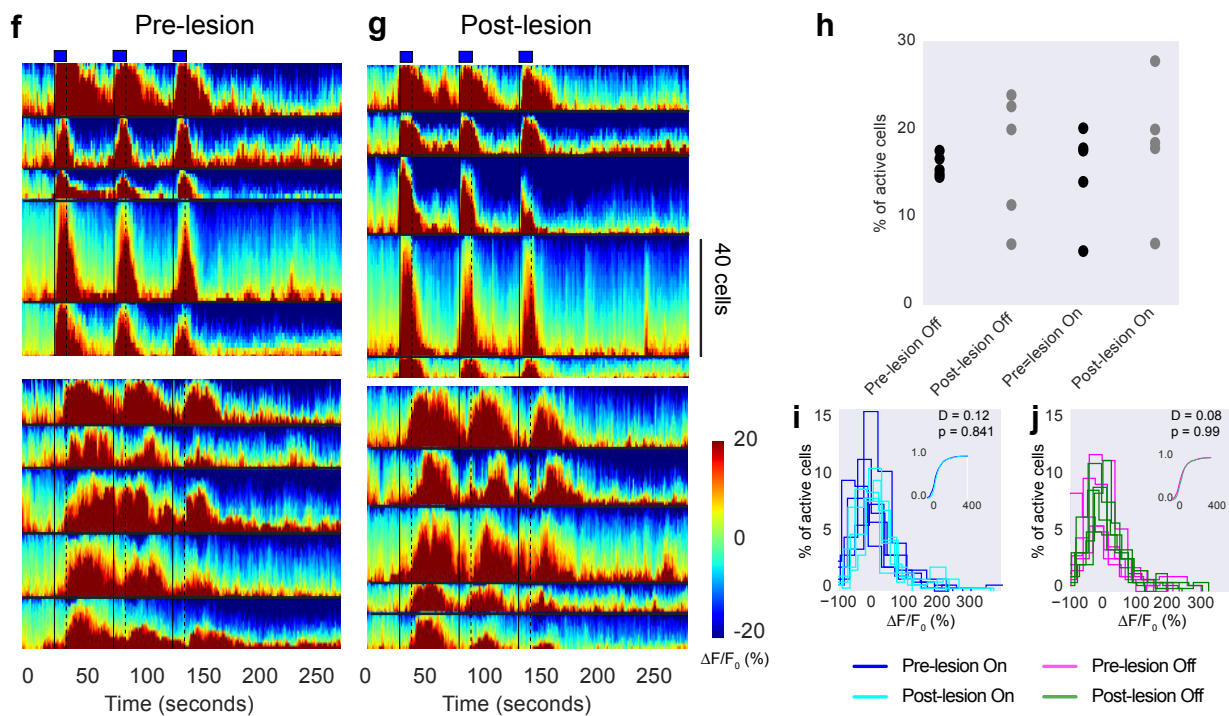

Supplement: Supplementary file 7 — The effect of the parapineal lesion on habenula response to blue light. a Visualization of the parapineal (yellow arrow), which is located adjacent to the left habenula and innervates the dorsal neuropil. b, c Two-photon lesioning of the parapineal. b Before lesioning. c After lesioning, which led to formation of a bubble (arrow). d, e Habenula cells segmented from five fish, overlaid on top of each other, showing responses before and after the lesion. Cells responding to light ON are shown in blue and to OFF in pink. f, g Heatmaps of the habenula cells, in the five fish, responding to light ON and OFF before (f) and after (g) lesioning the parapineal. Horizontal black lines divide data from different fish. h Percentage of cells showing ON and OFF responses before and after parapineal lesioning. i, j Histogram showing distribution of mean intensity in habenula neurons during light ON (e) and OFF (f) before and after lesion. Insets show cumulative distribution from all fish. P values and test statistic (D) were obtained using the Kolmogorov–Smirnov test. pp parapineal, lHb left habenula, rHb right habenula, cv circumventricular organ, a anterior, p posterior. Scale bar = 25 μm. (PDF 417 kb) [file 12915_2017_431_MOESM7_ESM.pdf]

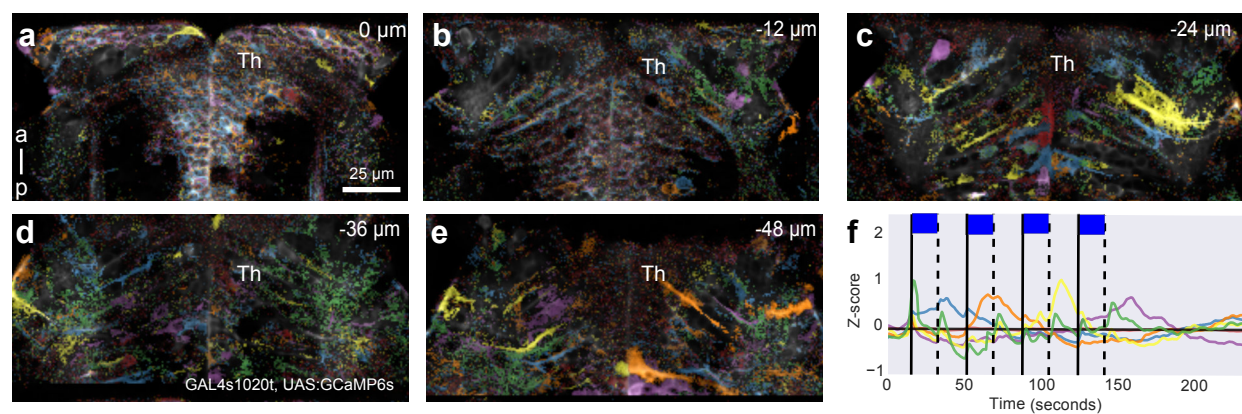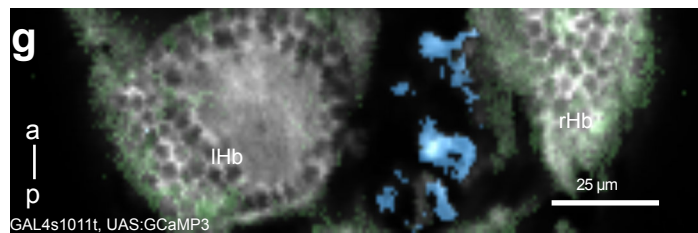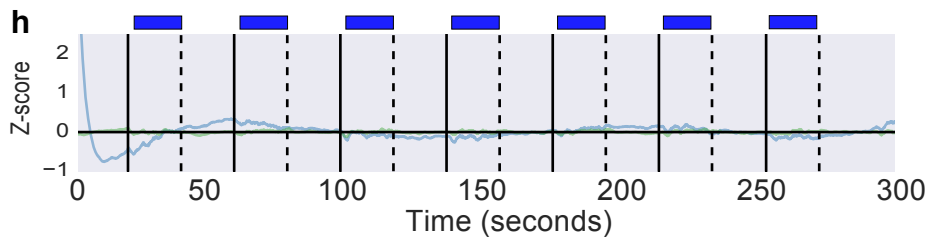

Supplement: Supplementary file 8 — Examples of signals that were excluded from visualization of K-means clusters. a–e Pixels showing stimulus-independent activity in the thalamus, at five different focal planes. Pixels are colored according to the traces in panel f. For clarity, these signals were excluded from the visualization of clusters representing light-evoked activity shown in Fig. 4a–e. g Stimulus-independent activity in the habenula. Pixels are colored according to the traces in panel h. For clarity, these signals were excluded from the visualization of clusters representing light-evoked activity shown in Fig. 1e–f. f, h Cluster centers that did not represent light-evoked activity in the thalamus and habenula, obtained by running K-means on the time series of pixels in panel a–e and g. Th Thalamus, lHb left habenula, rHb right habenula, a anterior, p posterior. (PDF 404 kb) [file 12915_2017_431_MOESM8_ESM.pdf]
